# Neonatal Abstinence Syndrome: A Case Series

**DOI:** 10.7759/cureus.64372

**Published:** 2024-07-11

**Authors:** Sudhir Malwade, Amulya Dharmagadda, Varsha Premkumar

**Affiliations:** 1 Pediatrics, Dr. D. Y. Patil Medical College, Hospital & Research Centre, Pune, IND

**Keywords:** psychotropics, finnegan, nicotine, heroin, phenobarbitone, pregnancy, drug abuse, neonate, abstinence

## Abstract

The increasing prevalence of substance misuse in modern culture is contributing to the growth in neonatal abstinence syndrome (NAS) cases in India. NAS can be challenging to diagnose due to nonspecific symptoms and maternal suppression of drug history. Only a few reports of NAS have been published from India. This is a case series of three newborns from India who all had symptoms like restlessness, high-pitched crying, excessive sweating, vigorous sucking, tremors, and diarrhea. The investigations did not lead to any conclusions. In the first case, the mother was treated with a combination of psychotropic medications, including selective serotonin reuptake inhibitors (SSRIs), atypical antipsychotics, and tricyclic antidepressants. In the second case, the mother was a nicotine addict, while in the third case, the mother had an opiate addiction. It was only after being asked several times that the abuse background of the last two cases was revealed. As a result, three cases of NAS were diagnosed, successfully managed with phenobarbitone, and discharged.

## Introduction

Neonatal abstinence syndrome (NAS), a postnatal drug withdrawal disorder, affects newborns and infants exposed to licit or illicit substances during the intrauterine period [1]. NAS is a multisystemic illness predominantly affecting the central, autonomic, and gastrointestinal systems. It can cause substantial neurodevelopmental morbidity in infants [2]. In India, the rate of drug abuse among women of reproductive age (ages 15 to 49) has risen dramatically over the last decade, posing a considerable challenge [3]. Even though there are numerous NAS case reports from around the world, there are not nearly as many from India due to a lack of disclosure of drug abuse and nonspecific disease symptoms. Because NAS diagnosis is primarily clinical, with or without biological testing [1], comprehensive history collection, and a high degree of suspicion are necessary for early detection. We present a case series of three infants diagnosed with NAS in our neonatal intensive care unit (NICU), successfully treated with phenobarbitone, and subsequently discharged.

## Case presentation

Case 1

A male newborn, born prematurely at 34 weeks, weighed 1.5 kg and was delivered by cesarean section. The Apgar score in the first and fifth minutes of life was six and seven, respectively. The mother, a 28-year-old primigravida, tested negative for human immunodeficiency virus (HIV), venereal diseases research laboratory (VDRL), and hepatitis B. Both parents are educated. The baby’s birth weight was 1.5 kg, which falls in the third percentile. The baby’s length was 46 cm, which is above the 50th percentile, and the head circumference was 31 cm, which is at the 50th percentile. The mother was well-built but anemic, with a hemoglobin level of 9 g/dl.

The mother’s history indicated that she had been diagnosed with a psychiatric illness for four years. Since then, she has been taking psychiatric medications such as imipramine, olanzapine, and sertraline during conception and pregnancy. The infant was immediately transferred to the NICU due to respiratory distress and prematurity. The baby had a Silverman Anderson Score (SAS) score of one on a scale of 10, so he was taken on oxygen by the hood and weaned off over the next two hours. The patient had a temperature of 37°C, a heart rate of 148/min, a respiratory rate of 66/min, a capillary filling time of less than three seconds, and a blood pressure of 86/48 (mean 60) mm Hg at admission. Subsequently, the infant showed an abnormal behavior pattern on the first day of life, which comprised excessive high-pitched crying, irritability, inconsolable crying, sweating, and tremors. The baby had an exaggerated Moro reflex and vigorous sucking. Based on these clinical symptoms, a differential diagnosis was made, considering sepsis along with meningitis, cerebral hemorrhage, hypocalcemia, hypoglycemia, and thyrotoxicosis. All the investigations performed turned out to be normal, creating a diagnostic conundrum.

Suspicion was directed toward NAS based on the history, clinical appearance, and normal laboratory profile. Symptomatic management was started for the patient with minimal stimuli, demand feeding, cuddling, kangaroo mother care, etc., and the Finnegan Scoring System (FSS) was meticulously monitored. Within 24 hours of the baby’s birth, three consecutive FSS scores showed values greater than eight. The maximum FSS score reached 15 on day three of life, and then phenobarbitone was started at 5 mg/kg/day, after which symptoms gradually decreased. Here is how we diagnosed the first case of newborn abstinence syndrome resulting from psychotropic medication withdrawal. This newborn has been discharged from the hospital after two weeks; treatment was continued totally for six weeks, and he is currently receiving ongoing care at our outpatient clinic for high-risk cases. The mother was recommended to continue taking her treatment for the illness.

Case 2

A male newborn weighing 2 kg was delivered by cesarean section at 33 weeks gestation due to placental abruption. The Apgar scores at one and five minutes after birth were six and seven, respectively. The mother was 35 years old, with one living child, and her HIV, VDRL, and hepatitis B status was negative. The parents had limited education. The baby’s birth weight was 2 kg, which falls within the 3rd percentile for gestational age. The baby’s length was 43 cm, above the 10th percentile, and the head circumference was 33 cm, at the 50th percentile. The mother was malnourished and anemic, with a hemoglobin level of 8.6 g/dl.

No history of addiction was disclosed during the initial questioning. Following birth, the baby was promptly sent to the NICU due to prematurity and respiratory distress. The newborn had a SAS score of three out of 10, so he was taken on continuous positive airway pressure (CPAP) and was given surfactant for hyaline membrane disease. Upon admission, the patient had a temperature of 36.5°C, a respiratory rate of 70/min, a heart rate of 152/min, a capillary filling time of less than three seconds, and a blood pressure of 68/36 (mean 48) mm of Hg. Hyaline membrane disease was resolved, and the respiratory condition improved. Subsequently, on the third day of life, the baby exhibited an atypical behavior pattern characterized by high-pitched crying, irritability, inconsolability, tremors, yawning, and gastrointestinal problems such as regurgitation, loose stools, and inadequate feeding. Based on these clinical symptoms, the possibility of sepsis with meningitis, cerebral hemorrhage, hypocalcemia, hypoglycemia, and enterocolitis was considered.

Relevant investigations were done, and all differential diagnoses were ruled out. Maintaining NAS as a differential, we initiated the maintenance of Finnegan scoring, which documented scores exceeding 15. After a second look at the history, it was found that the mother had been a nicotine addict for a few years. She smoked both during conception and throughout her pregnancy. Consequently, he was diagnosed with neonatal nicotine abstinence syndrome.

We initiated symptomatic treatment and started maintaining the Finnegan scoring system. Three consecutive FSS scores exceeding 15 and failed nonpharmacological therapy necessitated the initiation of phenobarbitone at a dosage of 5 mg/kg/day. Subsequently, symptoms progressively decreased, and the medication was gradually reduced and eventually discontinued over six weeks. The infant was discharged on the 10th day of life and is now being followed up in our high-risk outpatient clinic. The mother was referred to a rehabilitation and counseling center.

Case 3

A 38-week-old male newborn weighing 2.4 kg was delivered via normal delivery. The Apgar scores were seven and eight in the first and fifth minutes of life, respectively. Mother was a primigravida, 25 years old, and a registered case. Her HIV, VDRL, and hepatitis B status were negative. Parents were educated. The baby’s birth weight was 2.4 kg (10th percentile), his length was 47 cm (50th percentile), and his head circumference was 35 cm (90th percentile). Mother was malnourished and anemic with 7.9 g/dl hemoglobin.

The perinatal period was insignificant, and no NICU stays were documented. The infant was admitted to our hospital on the 17th day after birth due to symptoms like increased respiratory effort, inadequate sleep, frequent yawning, irritability, and a high-pitched cry. Upon admission, the patient presented with a fever of 37.0°C, a respiratory rate of 64 breaths per minute, a heart rate of 158 beats per minute, a capillary filling time of less than three seconds, and a blood pressure of 80/56 mmHg (mean 69 mmHg). The infant also exhibited strong sucking, greater muscle tone, and an exaggerated Moro reflex. Based on these clinical symptoms, the possibility of sepsis with meningitis, congenital heart disease, metabolic abnormalities, and thyrotoxicosis was considered.

Investigations indicated that the septic screen was negative, and the blood sugar and serum electrolytes were all within the normal range. The results of the chest X-ray and ultrasound of the skull did not reveal any abnormalities, and the thyroid function test was within normal limits. The results of the 2D echocardiogram were within normal limits. Subsequently, we started recording Finnegan scores, which continuously showed values exceeding eight for two consecutive days. A provocative history revealed the mother’s five-year heroin addiction, which had been initially hidden. She was taking the drug via inhalation. As a result, this led to the diagnosis of NAS in a third case involving heroin withdrawal.

After starting phenobarbitone at 5 mg/kg/day, symptoms subsided, and it was tapered over six weeks. The mother was referred to a rehabilitation center, and the infant was discharged and is being followed up in our high-risk clinic.

## Discussion

Globally, as of 2016, Australia and Asia had the highest prevalence of any substance use disorder, while North America had the highest prevalence of opioid dependence disorder [4]. The incidence of NAS has increased over the past few years from 4.6 to 6.7 per 1000 in-hospital births between 2012 and 2016 in the United States [5], and the national incidence of NAS increased from 3.4 to 5.8 (per 1000 hospital births) between 2009 and 2012, according to Indian health services. To our knowledge, Mishra et al. have documented a single case from India in 2010. The current article would mark the first case series involving three neonates diagnosed with NAS, each with a distinct cause [6].

Pregnant women have consumed more than 60% more prescription drugs in the last 30 years, with approximately 90% taking at least one and 70% taking a prescription drug [7]. Psychotropic medications are of particular concern, as they may differentially impact fetal neurodevelopment in comparison to other agents. Pocivalnik et al. documented a case of a neonate experiencing a severe, short-lived, inexplicable incident that was brought on by his mother’s consumption of sertraline during her pregnancy [8]. Certain parallels were found when comparing the two trials, like small for gestational age babies, normal Apgar scores, and the absence of perinatal events. In their study, a term neonate initially showed signs of a severe life-threatening event on day eight of life, such as an apneic episode accompanied by hypotonia and loss of consciousness, which was managed promptly. Subsequently, the neonate manifested additional symptoms. They excluded all other potential causes and diagnosed the neonate with NAS; however, nonpharmacological treatment resolved the condition. Contrary to this study, our patient was a premature newborn who required phenobarbitone despite presenting with mild to moderate symptoms within 24 hours after birth. In our study, this could be due to the maternal use of numerous classes of medications, including selective serotonin reuptake inhibitors (SSRIs), atypical antipsychotics, and tricyclic antidepressants. There are various studies on individual drug effects, but none on the combined effects of psychotropic drugs, and very little is known about them. This report represents the first documented case of NAS in India, resulting from the withdrawal of combined psychotropic medications. According to reports by Rowe et al. and Fukushima et al. neither our newborn had hypoglycemia nor prolonged QT syndrome as a result of the mother’s use of olanzapine and clomipramine (tricyclic antidepressants), respectively [9,10]. However, both our study and the study by Erol et al. [11], which diagnosed NAS due to prenatal citalopram exposure, showed a very similar course from presentation to treatment.

As per the National Family Health Survey-4 (2015-2016) and Global Adult Tobacco Survey-2 (2016-2017), approximately 5%-8% of expectant women are tobacco users. In neonatal nicotine abstinence syndrome, a variety of presentations are observed, ranging from common symptoms such as irritability, high-pitched crying, and tremors, as observed in our case and in a recent study by Nalesnik et al. [12], to adverse symptoms such as opisthotonos and fluctuations in muscular rigidity at 48 hours of life, as reported by Vagnarelli et al. [13]. Prenatal tobacco use has also been reported to result in adverse birth outcomes, including prematurity [14], low birth weight [15], and an exacerbation of NAS severity, which was also observed in our case.

In developed countries, opioid withdrawal is the most prevalent cause of NAS, and it is on the rise in developing countries such as India. The prevalence of current opioid use in the country is 2.06% [16]. The most frequently used opioid in India is heroin, with a prevalence of 1.14% [16]. The sole case of NAS reported from India to date is owing to heroin withdrawal, as reported by Mishra et al. [6]. Table 1 compares the findings of our case to those of the previously mentioned case. Our cases were identical in many respects, except for the fact that infants born to mothers who abuse heroin develop NAS immediately after birth. In our case, the neonate developed symptoms after 17 days of life, which was unusual. When opioids are administered concurrently with barbiturates or benzodiazepines, a delayed onset is observed.

**Table 1:** Comparison of both cases of neonatal abstinence syndrome due to heroin withdrawal Mishra et al. [6]

Parameter	Our case	Mishra et al. [6]
Gestational age	Term	Term
Birth weight	2.4 kgs ( at 10thpercentile)	2.2 kgs ( less than 10th percentile)
Day of presentation	Day 17 of life (unusual presentation)	Within 24 hours
Symptoms	Increased work of breathing, inadequate sleep, excessive yawning, irritability and high pitched cry	Fever, tachypnea, high-pitched crying, irritability, inconsolability, sweating, and diarrhea
Other causes	Not found	Not found
Finnegan score	Scores higher than eight	Scores higher than eight
Treatment	Not settled on nonpharmacological treatment, required phenobarbitone therapy	Not settled on nonpharmacological treatment, required phenobarbitone therapy

The Modified Finnegan Scoring System facilitates the initiation, monitoring, titration, and termination of therapy. Both nonpharmacological and pharmacological approaches are used in the management of NAS. At first, we tried demanding feeding, kangaroo mother care, cuddling, minimal stimulation, and swaddling. Despite all these trials, our Finnegan scores remained consistently high, suggesting that it is appropriate to initiate pharmacotherapy. The most often recommended medication is morphine, which also lowers the risk of seizures, improves feeding, controls agitation, and reduces diarrhea. The preferred medication for non-opioid withdrawal is primarily phenobarbitone. In cases of polydrug misuse, clinicians use it in conjunction with other drugs to manage severe symptoms. In all three of the cases we have discussed, we administered phenobarbitone for a total of six weeks and tapered it one month after discharge.

According to Kieviet et al. [17], breastfeeding is only prohibited while using illicit drugs. It is presumably protective against NAS. A multidisciplinary approach with parental participation is highly beneficial in managing these newborns, both during their hospital stay and after their discharge. Infants ought to undergo evaluations for neurodevelopment, growth, ophthalmology, psychobehavioral health, and family support during follow-up. All of our patients receive routine follow-up care in our high-risk clinic; thus far, none of them have experienced psychobehavioral problems or neurodevelopmental problems; instead, they are all thriving well.

## Conclusions

Given the rise in drug abuse in modern society, NAS is becoming an increasingly prevalent problem. Due to its widespread symptoms and covert history of drug misuse, it is frequently overlooked. To interpret the condition, one needs to consider that possibility. Regular follow-up is essential for early prediction and intervention since infants with NAS are at risk of long-term neurobehavioral and cognitive issues. To prevent child neglect and promote better development, parents must also focus on creating the best possible home environment.

## References

[1] Anbalagan S, Falkowitz DM, Mendez MD (Treasure Island (FL)). Neonatal Abstinence Syndrome. https://www.ncbi.nlm.nih.gov/books/NBK551498/.

[2] Merhar SL, McAllister JM, Wedig-Stevie KE, Klein AC, Meinzen-Derr J, Poindexter BB (2018). Retrospective review of neurodevelopmental outcomes in infants treated for neonatal abstinence syndrome. J Perinatol.

[3] Srivastava K, Rathod HK, Sharma P, Landage J, Vyas S, Banerjee A (2021). Substance use among females-study from rural Western India. Indian J Community Health.

[4] GBD 2016 Alcohol and Drug Use Collaborators (2018). The global burden of disease attributable to alcohol and drug use in 195 countries and territories, 1990-2016: a systematic analysis for the global burden of disease study 2016. Lancet Psychiatry.

[5] Strahan AE, Guy GP Jr, Bohm M, Frey M, Ko JY (2020). Neonatal abstinence syndrome incidence and health care costs in the United States, 2016. JAMA Pediatr.

[6] Mishra KK, Chopra N, Dudeja A, Datta V, Saili A, Dutta AK (2010). Neonatal abstinence syndrome. Kathmandu Univ Med J (KUMJ).

[7] Mitchell AA, Gilboa SM, Werler MM, Kelley KE, Louik C, Hernández-Díaz S (2011). Medication use during pregnancy, with particular focus on prescription drugs: 1976-2008. Am J Obstet Gynecol.

[8] Pocivalnik M, Danda M, Urlesberger B, Raith W (2018). Severe brief resolved unexplained event in a newborn infant in association with maternal sertralin treatment during pregnancy. Medicines (Basel).

[9] Rowe M, Gowda BA, Taylor D, Hannam S, Howard LM (2012). Neonatal hypoglycaemia following maternal olanzapine therapy during pregnancy: a case report. Ther Adv Psychopharmacol.

[10] Fukushima N, Nanao K, Fukushima H, Namera A, Miura M (2016). A neonatal prolonged QT syndrome due to maternal use of oral tricyclic antidepressants. Eur J Pediatr.

[11] Erol S, Ozcan B, Celik IH, Bas AY, Demirel N (2017). Neonatal abstinence syndrome due to prenatally citalopram exposure: a case report. Arch Argent Pediatr.

[12] Nalesnik G, Torine I (2024). A newborn with fever and tremors. Contemp Pediatr.

[13] Vagnarelli F, Amarri S, Scaravelli G, Pellegrini M, Garcia-Algar O, Pichini S (2006). TDM grand rounds: neonatal nicotine withdrawal syndrome in an infant prenatally and postnatally exposed to heavy cigarette smoke. Ther Drug Monit.

[14] Fantuzzi G, Aggazzotti G, Righi E (2007). Preterm delivery and exposure to active and passive smoking during pregnancy: a case-control study from Italy. Paediatr Perinat Epidemiol.

[15] Jaddoe VW, Troe EJ, Hofman A, Mackenbach JP, Moll HA, Steegers EA, Witteman JC (2008). Active and passive maternal smoking during pregnancy and the risks of low birthweight and preterm birth: the generation R study. Paediatr Perinat Epidemiol.

[16] Ambekar A, Agrawal A, Rao R, Mishra AK, Khandelwal SK, Chadda RK (2019). Magnitude of Substance Use in India. http://socialjustice.gov.in/writereaddata/UploadFile/Survey%20Report.pdf.

[17] Kieviet N, Dolman KM, Honig A (2013). The use of psychotropic medication during pregnancy: how about the newborn?. Neuropsychiatr Dis Treat.

